# Biocompatibility Analysis of the Silver-Coated Microporous Titanium Implants Manufactured with 3D-Printing Technology

**DOI:** 10.3390/nano14231876

**Published:** 2024-11-22

**Authors:** Maxim Shevtsov, Emil Pitkin, Stephanie E. Combs, Natalia Yudintceva, Denis Nazarov, Greg Van Der Meulen, Chris Preucil, Michael Akkaoui, Mark Pitkin

**Affiliations:** 1 Department of Radiation Oncology, Technische Universität München (TUM), Klinikum Rechts der Isar, Ismaninger Str. 22, 81675 Munich, Germany; stephanie.combs@tum.de; 2 Laboratory of Biomedical Nanotechnologies, Institute of Cytology of the Russian Academy of Sciences (RAS), 194064 Saint Petersburg, Russia; yudintceva@mail.ru; 3 Personalized Medicine Centre, Almazov National Medical Research Centre, 2 Akkuratova Str., 197341 Saint Petersburg, Russia; 4 Department of Statistics and Data Science, The Wharton School, University of Pennsylvania, Philadelphia, PA 19104, USA; emil.pitkin@gmail.com; 5 Saint Petersburg State University, Universitetskaya Nab, 7/9, 199034 Saint Petersburg, Russia; dennazar1@yandex.ru; 6 Movora, St. Augustine, FL 32095, USA; greg.vandermeulen@movora.com (G.V.D.M.); chris.preucil@movora.com (C.P.); 7 Tanury Industries, Lincoln, RI 02865, USA; michael@tanury.com; 8 Department of Orthopaedics and Rehabilitation Medicine, Tufts University School of Medicine, Boston, MA 02111, USA; 9 Poly-Orth International, Sharon, MA 02067, USA

**Keywords:** osseointegration, bone tissue engineering, fibroblasts, osteoblasts, scaffolds, titanium alloy, silver coating, 3D printing

## Abstract

3D-printed microporous titanium scaffolds enjoy good biointegration with the residuum’s soft and bone tissues, and they promote excellent biomechanical properties in attached prostheses. Implant-associated infection, however, remains a major clinical challenge. Silver-based implant coatings can potentially reduce bacterial growth and inhibit biofilm formation, thereby reducing the risk of periprosthetic infections. In the current study, a 1-µm thick silver coating was prepared on the surface of a 3D-printed microporous titanium alloy with physical vapor deposition (PVD), with a final silver content of 1.00 ± 02 mg/cm^2^. Cell viability was evaluated with an MTT assay of MC3T3-E1 osteoblasts and human dermal fibroblasts cultured on the surface of the implants, and showed low cytotoxicity for cells during the 14-day follow-up period. Quantitative real-time polymerase chain reaction (RT-PCR) analysis of the relative gene expression of the extracellular matrix components (fibronectin, vitronectin, type I collagen) and cell adhesion markers (α2, α5, αV, β1 integrins) in dermal fibroblasts showed that cell adhesion was not reduced by the silver coating of the microporous implants. An RT-PCR analysis of gene expression related to osteogenic differentiation, including TGF-β1, SMAD4, osteocalcin, osteopontin, and osteonectin in MC3T3-E1 osteoblasts, demonstrated that silver coating did not reduce the osteogenic activity of cells and, to the contrary, enhanced the activity of the TGF-β signaling pathway. For representative sample S5 on day 14, the gene expression levels were 7.15 ± 0.29 (osteonectin), 6.08 ± 0.12 (osteocalcin), and 11.19 ± 0.77 (osteopontin). In conclusion, the data indicate that the silver coating of the microporous titanium implants did not reduce the biointegrative or osteoinductive properties of the titanium scaffold, a finding that argues in favor of applying this coating in designing personalized osseointegrated implants.

## 1. Introduction

Silver-based coating of orthopedic devices is a promising approach for reducing postoperative infections. It effectively damages the bacterial cell membrane, increases its permeability, and reduces the likelihood of biofilm formation [1]. Indeed, silver coatings or silver-based nanoparticles exhibit antimicrobial activity against a broad spectrum of Gram-negative (Gr(−)) and Gram-positive (Gr(+)) bacteria, viruses, protozoa, and fungi [2,3]. In our previously published study, the silver coating applied via physical vapor deposition (PVD) increased the anti-bacterial properties of the titanium implant [4]. The PVD silver coating significantly reduced bacterial adhesion within 24 h. The coated implants measured 1.47 ± 0.21 × 10^6^ CFU (*S. aureus),* 1.47 ± 0.25 × 10^6^ CFU (*P. aeruginosa),* and 1.37 ± 0.25 × 10^5^ CFU (*S. epidermidis)*, compared to the non-treated control implants—4.23 ± 0.91 × 10^6^ CFU (*S. aureus*), 4.17 ± 0.75 × 10^6^ CFU (*P. aeruginosa*), 5.00 ± 0.53 × 10^5^ CFU (*S. epidermidis)* and (*p* < 0.001). Subsequent evaluation of bacterial planktonic growth also showed a significant reduction in the PVD silver-coated group [4]. The mechanisms of silver bactericidal properties include an increase in bacterial cell membrane permeability, oxidative damage via reactive oxygen species (ROS) production, disruption of DNA replication via ATP depletion, and inhibition of the bacteria’s respiratory chain and various enzymes [5,6,7]. Although several studies reported a low cytotoxicity profile toward normal cells and tissues while inhibiting bacterial growth, some reports demonstrated negative effects [8,9,10]. Indeed, Rosário et al. reported that for a certain size, silver nanoparticles (AgNPs) could either induce cell cycle arrest at the G0/G1 phase or apoptosis or necrosis in osteoblast-like MG-63 cells [9]. Silver-based coating cyto- and genotoxicity depends on time, dose, temperature, surface chemistry, and cell type, and should be taken into account when developing the design of implants, especially when using osseointegrated implants, the surface of which directly interacts not only with the soft tissues of the skin but also with bone tissue.

Another important effect of silver-based coatings is their promotion of osteogenesis and osteogenic differentiation [11,12,13,14,15,16,17]. Xie et al. reported that implants with a hybrid coating (containing chitosan, hydroxyapatite, and polydopamine) not only effectively inhibited biofilm formation for *S. aureus* (91.7%), *E. coli* (92.0%) and *S. epidermidis* (89.5%) but also enhanced osteogenic differentiation of MC3T3-E1 cells [11]. Furthermore, subsequent implantation of intramedullary nails into rat femurs enhanced bone-implant osseointegration (as shown by micro-CT examination and histological studies) [11]. In another study by Kuo et al., multilayer coatings that incorporated silver and strontium promoted osseointegration, angiogenesis, and antibacterial activity [12]. Genomic characterization employing RNA-seq technology showed that silver nanoparticles (AgNPs) enhanced differentiation and bone cell mineralization in MC3T3-E1 cells due to miRNA expression involved in the regulation of bone morphogenic proteins (including Bmp4, Bmp6, Fosl1) [18]. Another study by Cao et al. reported that AgNPs immobilized on titanium using the plasma immersion ion implantation process activated the MAPK/ERK signaling cascade via integrin α5, and this was associated with osteoblast differentiation in rat bone marrow stem cells [19]. Apart from activating osteogenic processes, AgNPs were shown to activate RhoA and induce actin polymerization [16]. MG-63 cell uptake of silver nanoparticles resulted in increased membrane penetrability, enhanced expressions of RANKL and Runx2, and decreased expressions of OCN, OPG, COL-1, and ALP [20].

In a recent study reported by our group, microporous 3D-printed implants showed good biocompatibility properties with strong fibroblast adhesion and growth, as well as MC3T3-E1 osteoblast osteogenic properties (see [21]). To impart antibacterial properties to the osseointegrated implants for subsequent preclinical translational studies, they were coated with a silver layer. In the present study, the possibly cytotoxic effect on dermal fibroblasts and MC3T3-E1 osteoblasts of silver-coated 3D-printed titanium implants was investigated. For human dermal fibroblasts, the expression of β1 integrin, α2 integrin (collagen-specific), α5 integrin (fibronectin-specific), αV integrin (vitronectin-specific), fibronectin, vitronectin, and collagen, and vitronectin genes was evaluated. For MC3T3-E1 cells, paxillin, vinculin, and FAK focal adhesion markers were estimated. Cell adhesion capacity, measured via the expression of certain adhesion molecules to the extracellular matrix (ECM) and cell/implant interface, determines cell growth and proliferation, which in turn is essential for the implant’s integration with surrounding skin and bone tissues [22]. Additionally, taking into account the possible pro-osteogenic effect of silver ions, osteogenic processes were studied during the co-incubation of MC3T3-E1 cells on silver-coated implants. For this, the expression of specific genes responsible for the processes of osteogenesis including osteonectin, osteocalcin, osteopontin, TGF-β1, and SMAD4 was evaluated [23]. Indeed, the TGF-β/BMPs signaling pathway has a widely recognized role in the bone formation and bone remodeling processes [24,25].

It was demonstrated that the silver-based coating has an acceptable cytotoxicity profile, while an expansion in the osteogenesis processes was observed.

## 2. Materials and Methods

### 2.1. Silver-Coated 3D-Printed Titanium Samples

The porous composite titanium experimental samples used in this in vitro study were 3D printed from medical-grade titanium alloy Ti6Al4V [26] at Movora, St. Augustine, FL 32095. The 3D printing process was developed to meet a patented combination of particle size, pore size, porosity, and volume fraction [27] that promotes deep ingrowth of surrounding tissues [28,29,30,31].

**Figure 1:**
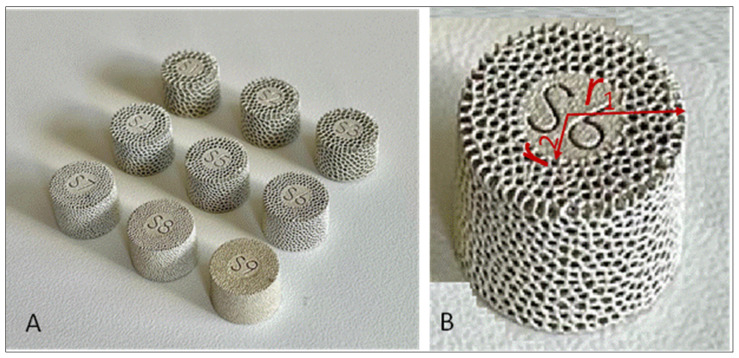
Tablets for the study: (**A**) set of tablets (S1–S9) fabricated with 3D-printing technology and coated with silver; (**B**) r_1_ is the outer radius of the tablets and r_2_ is the radius of a central solid core.

Samples were shaped as cylindrical tablets with a thickness of 10 mm, an outer radius (r_1_) of 6.8 mm, and a solid core radius (r_2_) of 3.4 mm, surrounded by porous cladding as in our recent study [21] (Figure 1).

### 2.2. 3D Printing vs. Sintering

In contrast with the experimental 3D-printed samples, the control samples were tablets fabricated with sintering technology, which is widely used in powered metallurgy and has various modifications [32,33,34]. The parameters of the samples were within the ranges specified in [27,35], animal studies on direct skeletal attachments of limb prostheses with skin-and-bone-integrated pylons [30,36,37] that had positive outcomes. The pylons were sintered from titanium (Ti6–AL–4V ELI) particles, and the solid inserts were made of Ti6–AL–4V ELI rods (Small-Parts, Seattle, WA, USA). Molds for sintering were machined from boron nitride (Momentive Performance Materials Inc., Strongsville, OH, USA). For each pylon, a solid bar was put into the mold and surrounded by titanium particles, creating a porous permeable body (cladding). The sintering proceeded for 2 h at a temperature of 1190 °C followed by capsule-free hot isostatic pressure (HIP) treatment [38]. The simultaneous application of heat and pressure in the inert gas atmosphere eliminated internal voids and microporosity and improved the consolidation of powder metals and metal cladding after sintering.

The composed part of the pylon needs to be implanted post-amputation into the bone remnant of the residuum of the animal leg; the outside portion of the solid bar is used for attachment of the leg prosthesis. However, the bond between the solid bar and the porous cladding was not strong enough under rough spike loadings during the animals’ ambulation. Therefore, in a recent study [39] we began exploring the replacement of the sintering technology by 3D printing, considering its important advantages in the greater intrinsic strength of the composed implant, and because it enables the implant to be fabricated with different customized structures of porous cladding to interface with the hosting residuum’s bone and skin.

### 2.3. Specifics of 3D Printing Fabrication

The porous titanium implants were initially designed in Solidworks (Dassault Systèmes, GSC, Germantown, WI, USA) to quantify the porous and solid bodies of each implant. The designed constructs were next uploaded into the 3-Matic program (Materialise, Leuven, Belgium) as an assembly, and different porous bodies with varying lattices were constructed. The models of the completed porous Ti implants were uploaded into the Magics (Materialise, Leuven, Belgium) program to further design the layout and subsequently slice the files for 3D printing. The generated file was imported into an M2 Series 5 printer (Colibrium Additive, Rock Hill, SC, USA), and the Ti samples were printed.

- Type of additive printing process, DMLM.
- Parameters used for 3D printing-Mesh+
- Slice thickness for generating build file-30 microns

Following removal from the build plate employing a Wire EDM (Wire Discharge Machining, Waukesha, WI, USA), each of the Ti samples was bead blasted (glass bead—150–212 µm, 40–60 psi) in order to remove any unsolidified powder, and then ultrasonically cleaned. A set (S1–S9) of 12 tablets for each sample was fabricated with an average pore size of 210–1000 µm (Table 1).

### 2.4. Specifics of Silver Coating of the Samples

After cleaning, tablets were coated with a 1-µm silver layer with an intermittent pattern (Tanury Industries, Lincoln, RI, USA) as patented in [27] and previously reported in [4,36]. The technology used was physical vapor deposition (PVD) [40], and the equipment used was the magnetron sputtering multi-target machine, Flexicoat series (IHI Hauzer Techno Coating B.V., Venlo, The Netherlands). The silver content of the test items was 1.00 ± 0.2 mg/cm^2^, and the total silver content per test item was 5.0 ± 0.1 mg.

Since the implantation of pylons in DSA patients is permanent, this coating specification was selected to combine the well-established bactericidal properties of silver [41] with a relatively fast dissolution of the silver layer, in order to avoid the toxic consequences of long-term exposure to silver [42,43,44,45,46,47]. Positive verification of this specification was reported in our animal studies [4,36] where the skin-and-bone-integrated pylons (SBIPs) had a silver layer thin enough to dissolve within about 4–6 weeks after implantation. That period was sufficient for the skin to regenerate into the porous cladding of the SBIP pylon and establish a sustainable natural barrier against infection.

### 2.5. Cells

Human dermal fibroblasts were grown in a DMEM medium supplemented with 6  mM L-glutamine, 0.1  mM MEM non-essential amino acids, 4.5  g/L glucose, 1  mM MEM sodium pyruvate, 10% fetal bovine serum (FBS), and 1% antibiotics penicillin-streptomycin (Gibco, Waltham, MA, USA). Mouse MC3T3-E1 (ATCC, CRL-2594) cells were grown in an α-minimum essential medium (α-MEM) (Gibco, Waltham, MA, USA) supplemented with  6 mM L-glutamine, 0.1  mM MEM non-essential amino acids, 4.5  g/L glucose, 1  mM MEM sodium pyruvate, 10% (FBS) (Gibco, Waltham, MA, USA) and 1% antibiotics Pen/Strep (Gibco, Waltham, MA, USA). All cells were cultured at 37 °C and 5% CO_2_. To evaluate the influence of silver coating on the osteogenic activity of cells grown on the Ti implants, MC3T3-E1 cells were grown in an osteoinductive medium α-MEM supplemented with 50 μg/mL of l-ascorbic acid (Sigma-Aldrich, St. Louis, MA, USA), 10 mM β-glycerophosphate (Sigma-Aldrich, St. Louis, MA, USA), and dexamethasone (100 nM) (Sigma-Aldrich, St. Louis, MA, USA).

### 2.6. MTT Assay

The proliferation of MC3T3-E1 cells and human dermal fibroblasts was evaluated by a 3-[4,5-dimethylthiazol]-2, 5-diphenylterazolium bromide assay (MTT assay) (Invitrogen, Waltham, MA, USA). In brief, cells were grown in 96-well plates at a density of 5 × 10^3^ cells/well. After the silver-coated Ti samples were seeded with cells, the implants were co-incubated at 37 °C, 5% CO_2_ for 1, 3, 7, and 14 days. At the end of the incubation period, 20 μL of MTT (0.5 mg/mL) solution was added to each well and incubated at 37 °C for 4 h. The 96-well plate was assessed at 490 nm with a microplate reader (Bio-Rad, Hercules, CA, USA, model 550). All experiments were performed in triplication independently. Proliferation cell rate (%) = (sample OD − blank OD)/(control OD − blank OD) × 100%.

### 2.7. Scanning Electron Microscopy

Prior to scanning electron microscopy (SEM) analysis, the samples were subjected to autoclavation. Subsequently, the samples were inoculated with MC3T3-E1 cells and fibroblasts (5 × 10^6^/mL) on their surface for 72 h in a CO_2_ incubator. Subsequently, 300 µL of nutrient medium was added to each well, thereby ensuring that the surface of the samples was completely covered. Subsequently, the cells were washed with Dulbecco’s phosphate buffer saline (PBS) (Sigma-Aldrich, USA) and fixed in 2.5% glutaraldehyde in phosphate buffer (pH = 7.2, Sigma-Aldrich, USA). Following a three-day incubation period, the samples were washed in phosphate buffer (pH = 7.2, Sigma-Aldrich, USA) and successively dehydrated in 30, 50, 70, 90, 96%, and absolute ethanol (30 min each). The final drying process was conducted three times for 15 min using the Leica EM CPD300 at the CO₂ critical point. Subsequently, the conductive silver coatings, with an approximate thickness of 10 nm, were deposited using the Leica EM SCD500. The morphology of the cells was evaluated using a scanning electron microscope Zeiss Auriga (Carl Zeiss, Oberkochen, Germany), employing SE (secondary electrons) and in-lens regimes at magnifications ranging from 300 to 10,000.

### 2.8. Real-Time PCR Analysis

Total extracted RNA from MC3T3-E1 osteoblasts and dermal fibroblasts grown on silver-coated Ti implants at each time point was obtained with the Qiagen RNA Plus kit (QIAGEN, Venlo, Netherlands) and total RNA was quantified with a nanodrop spectrophotometer (Thermo Scientific, Waltham, MA, USA). In brief, cDNA synthesis for subsequent real-time PCR experiments used the superscript III reverse transcriptase (RT) enzyme (Invitrogen, Waltham, MA, USA). A reaction mix (containing 50 μM oligodT and 10 mM deoxyribonucleotide triphosphate mix (dNTP)) was added to 2 μg RNA for first strand synthesis at 65 °C for 5 min with subsequent cooling at +4 °C on ice for 2–3 min. Then a mix containing Superscript III reverse transcriptase, 0.1 M DTT (Di-thio-threitol), RNase inhibitor, and 5× reaction buffer was added to the first strand synthesized mixture with subsequent incubation at 50 °C for 1 h and followed by inactivation of RT at 70 °C for 15 min. As a control for calculating fold differences in RNA levels of MC3T3-E1 osteoblasts and fibroblasts grown on silver-coated implants, cDNA for GAPDH was used. PubMed nucleotide design (Primer-BLAST) software was used for the design of forward and reverse primer-specific genes (Table 2). Following the manufacturer’s protocol, the obtained samples were assessed in the Applied Biosystems 7900HT Fast real-time PCR system (Applied Biosystems, Waltham, MA, USA).

### 2.9. Statistical Analysis

We repeated measurements for each biomarker and extracellular matrix component over multiple time periods (2 or 4 times, depending on which one). Each of these measurements was performed three times independently on each of the experimental discs S1–S9 and means and standard deviations were computed in Excel. For the MC3T3 and dermal fibroblast measurements, a blank control, sintered Ti sample, and 3D-printed sintered Ti sample were also used. Bar charts were created for every marker/tablet/time period combination, with the average of the three measurements the height, and ±1 SD error bars overlayed. To compare expression at the end of the incubation period, a one-way ANOVA (test for equality of means) was run for each marker for the last time period. The independent variable was the disc number. We assumed equality of within-group variances and posted a significance level of alpha = 0.05. We used the post hoc Tukey–Kramer test to ascertain whether specific discs or groups of discs were significantly different whenever significant differences in means were detected by the ANOVA test.

## 3. Results

### 3.1. Analysis of Cell Viability and Adhesion on the Silver-Coated 3D-Printed Titanium Implants

**Figure 2:**
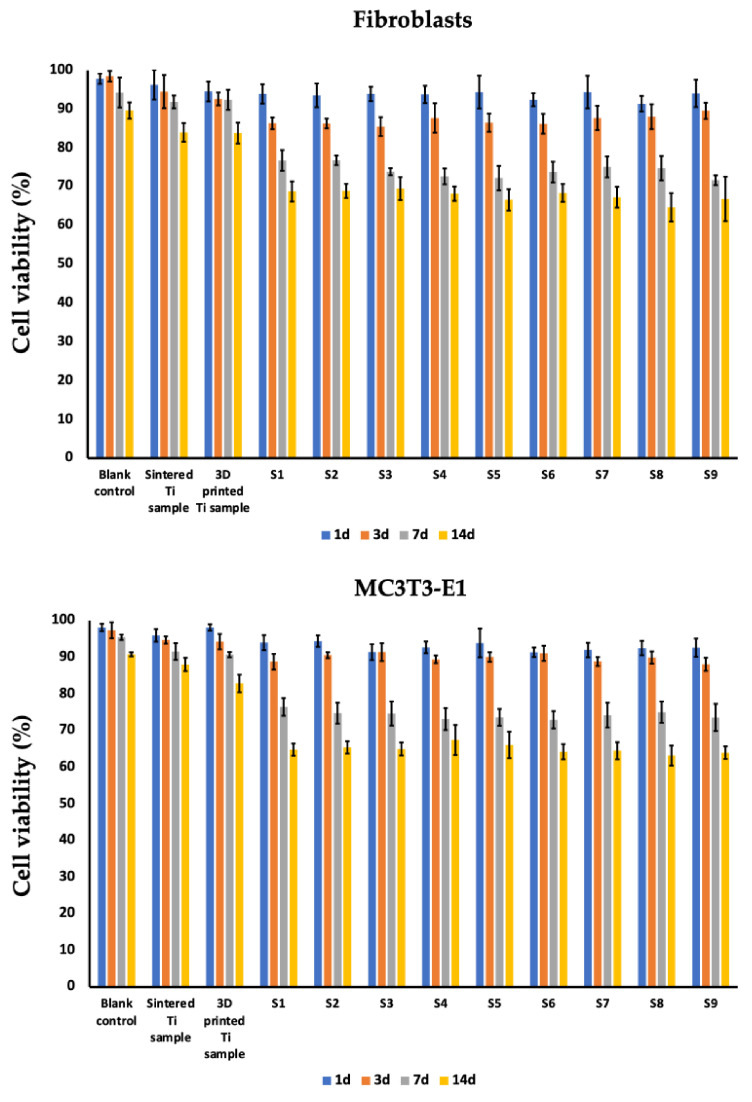
MTT assay of dermal fibroblasts and MC3T3-E1 osteoblast cells on silver-coated 3D-printed titanium microporous implants (S1–S9). Cell viability (%) was evaluated on the 1st, 3rd, 7th, and 14th day after co-incubation. Sintered Ti implant and 3D-printed implant without silver coating were used as controls. Data is presented from three independent experiments as M ± SD.

**Figure 3:**
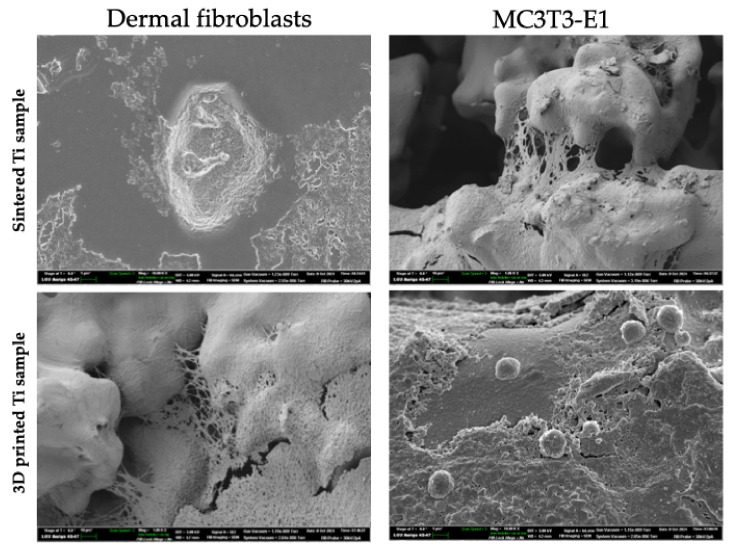
Representative scanning electron microscopy images of MC3T3-E1 cells and fibroblasts cultured on the samples S5 following 72 h of co-incubation.

Dermal fibroblasts and MC3T3-E1 osteoblast viability were evaluated in vitro with an MTT assay where cells were grown on silver-coated 3D-printed titanium implants with various pore sizes (S1–S9) for 1, 3, 7, and 14 days (Figure 2, Table 3). In the negative control group (blank control), when both cell types were grown in cultural flasks without implants, the cell viability (%) for dermal fibroblasts on the 1st, 3rd, 7th, and 14th days was 97.83 ± 1.31%, 98.5 ± 1.35%, 94.3 ± 3.86%, and 89.63 ± 2.1%, respectively. The cell viability (%) for MC3T3-E1 osteoblasts on the 1st, 3rd, 7th, and 14th days of culturing in flasks as a monolayer culture was 98.13 ± 1.0%, 97.37 ± 2.17%, 95.47 ± 0.74%, and 90.8 ± 0.56%, respectively. When these cells were cultured either on sintered titanium or 3D-printed titanium implants without silver coating, no significant decrease in cell viability was detected in the follow-up period of 14 days (Figure 2, Table 3). However, when cells were grown on the surface of silver-coated implants, a decrease in cell viability was observed. Accordingly, for the dermal fibroblasts cultured on the representative S1 sample on the 1st, 3rd, 7th, and 14th days, viability was 93.93 ± 2.50%, 86.37 ± 1.50%, 76.77 ± 2.68%, and 68.77 ± 2.59%, respectively. For MC3T3-E1 osteoblasts cultured on the representative S1 sample on the 1st, 3rd, 7th, and 14th days, viability was 94.0 ± 2.07%, 88.77 ± 2.14%, 76.43 ± 2.40%, and 64.77 ± 1.67%, respectively. When the viability for both cell types was compared between the samples S1–S9 no statistically significant difference (*p* > 0.05) was observed. Additionally, cell adhesion of both MC3T3-E1 cells and fibroblasts was evaluated employing SEM studies. Thus, following 72 h after seeding of the cells on the samples S1–S9 we detected the formation of the cells monolayer and cells spreading in all tested samples (Figure 3).

### 3.2. Evaluation of Cell Adhesion Markers Expression for MC3T3-E1 Osteoblasts and Dermal Fibroblasts Grown on Silver-Coated 3D-Printed Titanium Implants

**Figure 4:**
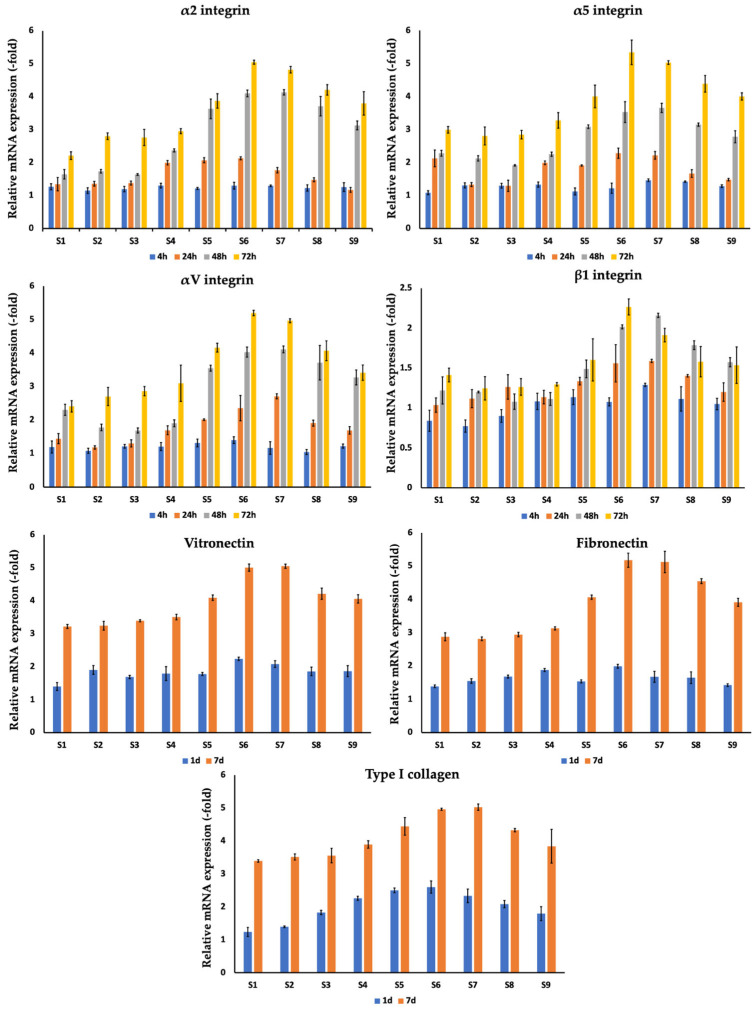
Comparison of expression of integrins and extracellular matrix component (fibronectin, vitronectin, type I collagen) genes of dermal fibroblasts on silver-coated 3D-printed titanium implants S1–S9 4, 24, 48, and 72 h after co-culturing. Analysis of gene expression related to fibronectin, vitronectin, and type I collagen was performed following 4 and 72 h of co-culturing cells on the surface of implants. Data is presented from three independent experiments as M ± SD. *p* < 0.01 for testing mean expression levels.

Following evaluation of cell viability (%), expression of genes related to cellular adhesion (α2 integrin (collagen-specific), α5 integrin (fibronectin-specific), αV integrin (vitronectin-specific), and β1 integrin genes) was assessed for dermal fibroblasts 4, 24, 48, and 72 h after co-incubation on the silver-coated 3D-printed titanium implants (Figure 4, Table 4). For all evaluated samples S1–S9, a gradual increase in gene expression was observed during the 72-h follow-up period. Of note, the highest values of the studied gene expressions were detected for the S5–S9 implants (diameter of pores ranging from 590 to 210 µm), reaching its peak for sample S6 with a subsequent decrease for the S9 sample. For the representative S6 sample (500 µm pores diameter), following 72 h of co-culture, the values were 2.27 ± 0.1 (β1 integrin), 5.05 ± 0.06 (α2 integrin), 5.34 ± 0.37 (α5 integrin), and 5.2 ± 0.08 (αV integrin). Evaluation of gene expression related to fibronectin, vitronectin, and type I collagen was performed at 4 and 72 h after co-culturing fibroblasts on the surface of 3D implants (Figure 4, Table 4). As for the previously described markers, the best gene expression values were observed in the sample line S5–S9 with the highest values for sample S6, which after 72 h were 5.18 ± 0.21 (fibronectin), 4.96 ± 0.03 (collagen), 5.0 ± 0.11 (vitronectin).

**Figure 5:**
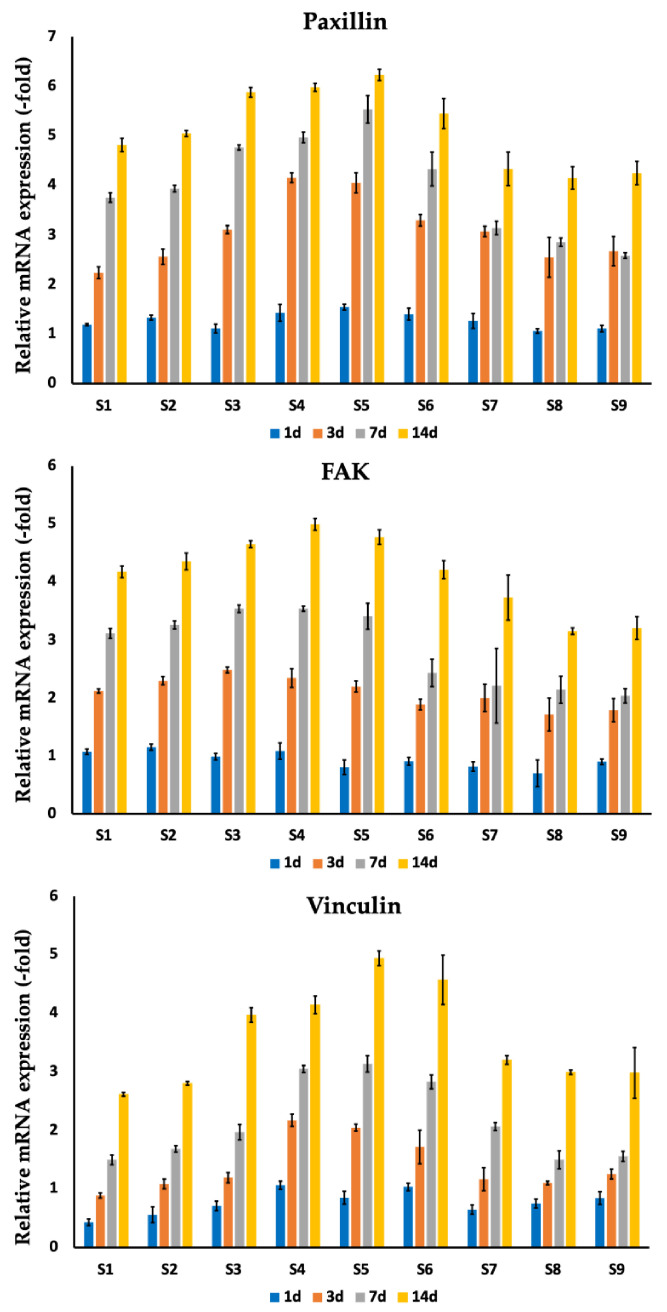
Comparison of gene (FAK, vinculin, paxillin) expression for MC3T3-E1 cells co-cultured on silver-coated titanium implants with various pore sizes (S1–S9) after 1, 3, 7, and 14 days. Data is presented from three independent experiments as M ± SD. *p* < 0.01 for testing mean expression levels.

Additionally, for MC3T3-E1 osteoblast cells, adhesion markers (FAK, vinculin, and paxillin) were evaluated following 1, 3, 7, and 14 days of co-culturing on silver-coated implants (S1–S9) (Figure 5, Table 5). For all samples, a gradual increase in gene expression was detected. The best gene expression values were observed in the sample line S3–S7 (diameter of pores ranging from 770 to 420 µm) with the highest values for samples S4 and S5, which after 14 days were 5.0 ± 0.1 (FAK), 5.98 ± 0.08 (paxillin), 4.15 ± 0.15 (vinculin) for S4 and 4.78 ± 0.13 (FAK), 6.23 ± 0.11 (paxillin), 4.94 ± 0.42 (vinculin) for S5.

### 3.3. Evaluation of the Genes Expression Related to Osteogenic Processes for MC3T3-E1 Osteoblast Cells Cultured on Silver-Coated 3D-Printed Titanium Implants

**Figure 6:**
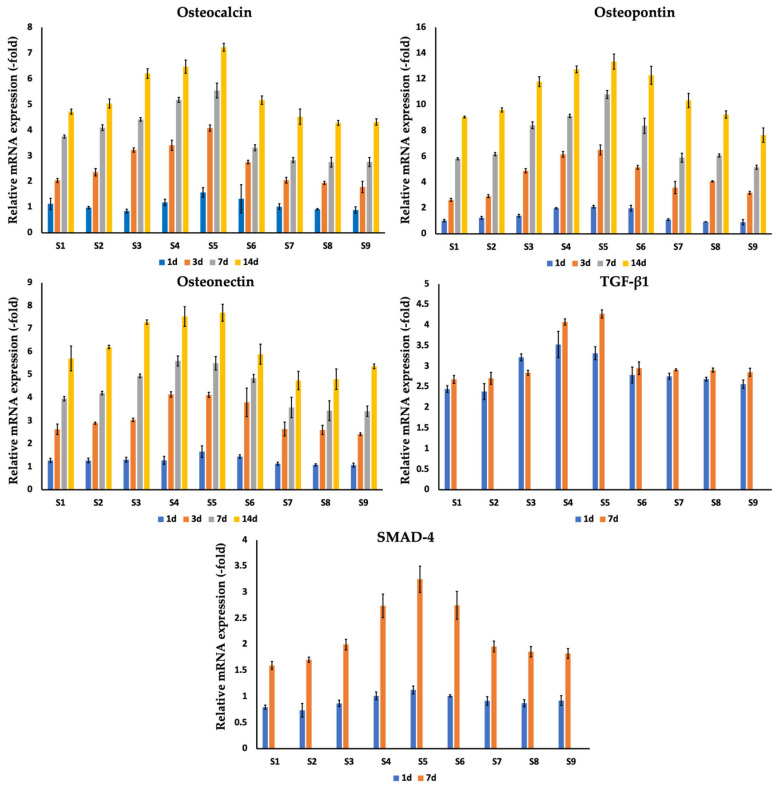
Comparison of expression of osteocalcin, osteopontin, and osteocalcin genes of MC3T3-E1 cells following co-incubation with silver-coated titanium implants (S1–S9) after 1, 3, 7, and 14 days. Analysis of TGF-β1 and SMAD4 gene expression in MC3T3-E1 osteoblast cells was performed on days 1 and 7 after co-incubation. Data is presented from three independent experiments as M ± SD. *p* < 0.01 for testing mean expression levels.

To assess osteogenic processes in MC3T3-E1 osteoblast cells, osteocalcin, osteopontin, and osteonectin gene expression was evaluated on the 1st, 3rd, 7th, and 14th day of co-incubation on silver-coated implants (Figure 6, Table 6). For all samples, a gradual increase in all studied gene expressions was observed over the follow-up period of 14 days. The highest values were detected for samples S4–S6, reaching peak values for sample S5 at day 14, of 7.22 ± 0.15 (osteocalcin), 13.35 ± 0.59 (osteopontin), 7.7 ± 0.37 (osteonectin). Additionally, TGF-β1 and SMAD4 gene expression analysis was performed on days 1 and 7 after co-incubation. All studied samples S1–S9 showed a gradual increase of TGF-β1 and SMAD4 gene expression The highest values were detected for samples S4–S6, reaching the peak values for sample S5 at day 7 of 4.27 ± 0.1 (TGF-β1), 3.25 ± 0.25 (SMAD4).

## 4. Discussion

The study and implementation of new antibacterial coatings for osseointegrated implants is one of the important areas of translational orthopedics and traumatology [48]. One of the most common approaches to coatings relies on various metals (e.g., silver, copper, zinc, etc.) [49,50,51,52]. In this study, silver was chosen for a coating because of its antibacterial properties, potential toxicity, the possibility of translation into clinical practice, high efficiency, and low cost. Our data showed tolerable cytotoxicity of the silver-coated 3D-printed implants and are in line with previously reported in vitro and in vivo studies that demonstrated low cytotoxicity or implant-related side effects [2,53,54]. In a recent clinical study reported by Savvidou et al., knee arthrodesis with a silver-coated implant in eight patients was associated with eradication of infection and good clinical performance [55]. Previously reported in vitro studies were largely based on very high concentrations of silver-based coatings or nanoparticle exposure for short periods of time (hours, days), which does not correspond to in situ microenvironment exposure or Ag^+^ clearance by blood and lymph microcirculation. Thus, in our group’s study employing dispersive X-ray spectroscopy in analyzing silver release, we showed trace amounts of silver after 3 months of in-bone intramedullary insertion of silver-coated implants into the femur of New Zealand rabbits [36]. In another set of experiments using pig and rabbit dorsum models to evaluate skin ingrowth into the micropores of the implant, no postoperative complications were reported at the end of the follow-up period of 6 months (as supported by histological studies) [36]. It can be assumed that the observed cytotoxicity of silver coatings according to the MTT test (Figure 2, Table 3) will be significantly lower in in vivo studies due to the rapid degradation of the silver coating under the influence of the tissue microenvironment.

Another approach to decrease periprosthetic infections could be based on drug-releasing implant coatings [56]. In a recent study, Ghimire et al. reported the efficient application of poly(ethylene glycol) dimethacrylate hydrogel coatings with vancomycin (PEGDMA-Oligo-Vanco) of implants, when the antibiotic was released into the surrounding tissues by the cleavage of an oligonucleotide (Oligo) linker by micrococcal nuclease (MN) secreted by *S. aureus* [57]. Indeed, intramedullar insertion of Ti6Al4V pins coated with PEGDMA-Oligo-Vanco) into mouse femurs with subsequent inoculation of *S. aureus* effectively prevented periprosthetic infection and sustained bacterial clearance [57,58]. In a more recent study, the authors, employing this coating platform, used β-lactam antibiotic ampicillin which has a broad spectrum and also affects Gr(–) bacteria [59]. Presumably, a combination of silver-coated SBIP implants with certain drug-releasing coatings will increase the anti-bacterial potential of the implant but this hypothesis should be tested in a separate experimental set.

Apart from antimicrobial peptides and/or antibiotics, other organic materials could also be employed (reviewed in [60]). Several studies recently showed the antibacterial efficacy of novel agents (e.g., red phosphorus (P)/IR780/arginine-glycine-aspartic acid-cysteine (RGDC), 5-(4-bromophenyl)-N-cyclopentyl-1-octyl-1H-imidazol-2-amine (LC0024), N-alkylated 3,6-dihalocarbazole 1-(sec-butylamino)-3-(3,6-dichloro-9H-carbazol-9-yl) propan-2-ol (SPI031)) that additionally did not affect the proliferation of attached cells or osseointegrative processes [61,62,63].

## 5. Conclusions

Due to their high anti-bacterial efficiency and low cost, silver-based coatings are widely used in reducing periprosthetic infectious complications in orthopedics and traumatology. In the current study, silver-coated microporous titanium implants demonstrated a low cytotoxicity profile combined with good biocompatibility, as illustrated on dermal fibroblasts and MC3T3-E1 osteoblast cells. Meanwhile, the osteogenic activity of MC3T3-E1 cells was preserved (as shown by TGF-β1 and SMAD4 gene expression). The data can be employed for follow-up in vivo preclinical studies for the design of transcutaneous osseointegrated titanium implants.

## Figures and Tables

**Table 1:** Characteristics of the 3D-printed silver-coated Ti samples. The designed pore sizes and strut diameters were obtained using a Dino-Lite Camera (magnification of 50.6) through an average of five measurements. Sample identifier (SI) refers to the number that was engraved on each different Ti implant. % Porous parameter represents the percentage (%) of the total volume of the lattice section of the Ti implant that is void of material. % Solid parameter is the percentage (%) of the total volume of the lattice structure that is titanium (Ti) metal. Strut diameter (µm) represents the diameter of the lattice beams. Average pore diameter (µm) refers to the average diameter of a sphere that lies tangent to the surrounding lattice beams.

Sample Identifier (SI)	% Porous	% Solid	Average Pore Diameter (µm)	Strut Diameter (µm)
S1	71.4	28.6	1000	270
S2	67.3	32.7	890	270
S3	62.7	37.3	770	270
S4	56.8	43.2	690	260
S5	50.0	50.0	590	270
S6	41.3	58.7	500	270
S7	21.2	78.8	420	250
S8	31.1	68.9	310	250
S9	19.5	80.5	210	230

**Table 2:** Forward and reverse primers designed for real-time PCR analysis.

Gene	Primers (5′–3′)	Product Length (bp)
α2 integrin	Fwd AAGTGCCCTGTGGACCTACCCA Rev TGGTGAGGGTCAATCCCAGGCT	119
α5 integrin	Fwd ACCACCTGCAGAAACGAGAGGC Rev TGGCCCAAACTCACAGCGCA	111
αV integrin	Fwd TCCCACCGCAGGCTGACTTCAT Rev TCGGGTTTCCAAGGTCGCACAC	121
β1 integrin	Fwd TTCAGACTTCCGCATTGGCT Rev AATGGGCTGGTGCAGTTTTG	122
Fibronectin	Fwd TGCAGTGGCTGAAGTCGCAAGG Rev GGGCTCCCCGTTTGAATTGCCA	119
Vitronectin	Fwd TGTTGATGCAGCGTTCGCCCT Rev TCCTGGCTGGGTTGCTGCTGAA	114
Type I collagen	Fwd CTCCTGACGCATGGCCAAGAA Rev TCAAGCATACCTCGGGTTTCCA	100
Vinculin	Fwd TCAAGCTGTTGGCAGTAGCCGC Rev TCTCTGCTGTGGCTCCAAGCCT	120
FAK	Fwd AGCACCTGGCCACCTAAGCAAC Rev CATTGGACCGGTCAAGGTTGGCA	125
Paxillin	Fwd AGGGCCTGGAACAGAGAGTGGA Rev AGCTGCTCCCAGTTTTCCCCTG	129
TGF-β1	Fwd ACCCGCGTGCTAATGGTGGA Rev GGGCACTGCTTCCCGAATGTCT	111
SMAD4	Fwd AGCCAGGACAGCAGCAGAATGGA Rev ATGGCCGTTTTGGTGGTGAGGC	128
Osteocalcin	Fwd AGCAGGAGGGCAATAAGGTAGT Rev TCGTCACAAGCAGGGTTAAGC	118
Osteonectin	Fwd ATGTCCTGGTCACCTTGTACGA Rev TCCAGGCGCTTCTCATTCTCAT	103
Osteopontin	Fwd TGATTCTGGCAGCTCAGAGGA Rev CATTCTGTGGCGCAAGGAGATT	110

**Table 3:** Mean (with standard deviation) cell viability (%) of dermal fibroblasts and MC3T3-E1 osteoblast cells on silver-coated 3D-printed titanium microporous implants (S1–S9). Cell viability (%) was evaluated on the 1st, 3rd, 7th, and 14th day after co-incubation. Sintered Ti implant and 3D-printed implant without silver coating were used as controls.

**MC3T3-E1 Osteoblasts**												
	**Blank Control**	**Sintered** **Ti Sample**	**3D-Printed ** **Ti Sample**	**S1**	**S2**	**S3**	**S4**	**S5**	**S6**	**S7**	**S8**	**S9**
**1 d**	98.13 (1)	95.97 (1.7)	98.13 (0.85)	94 (2.07)	94.43 (1.57)	91.4 (2.17)	92.7 (1.61)	93.9 (3.93)	91.37 (1.31)	91.97 (1.99)	92.5 (1.97)	92.63 (2.48)
**3 d**	97.37 (2.17)	94.7 (0.98)	94.27 (2.1)	88.77 (2.14)	90.57 (0.81)	91.4 (2.43)	89.43 (1.03)	90.07 (1.31)	91.07 (2.06)	88.83 (1.21)	89.93 (1.65)	88.07 (1.78)
**7 d**	95.47 (0.74)	91.57 (2.28)	90.7 (0.72)	76.43 (2.4)	74.73 (2.85)	74.63 (3.29)	73.1 (2.99)	73.6 (2.29)	72.93 (2.38)	74.2 (3.39)	75 (2.91)	73.57 (3.7)
**14 d**	90.8 (0.56)	88.03 (1.8)	82.83 (2.4)	64.77 (1.67)	65.37 (1.68)	64.97 (1.8)	67.4 (4.09)	66.03 (3.61)	64.2 (2.09)	64.47 (2.35)	63.13 (2.75)	63.93 (1.72)
**Dermal Fibroblasts**												
	**Blanc Control**	**Sintered ** **Ti Sample**	**3D-Printed ** **Ti Sample**	**S1**	**S2**	**S3**	**S4**	**S5**	**S6**	**S7**	**S8**	**S9**
**1 d**	97.83 (1.31)	96.3 (3.83)	94.57 (2.57)	93.93 (2.5)	93.57 (3.07)	93.93 (1.85)	93.8 (2.23)	94.4 (4.23)	92.43 (1.68)	94.4 (4.2)	91.4 (2.02)	94.07 (3.52)
**3 d**	98.5 (1.35)	94.53 (4.28)	92.63 (1.66)	86.37 (1.5)	86.3 (1.25)	85.5 (2.43)	87.73 (3.8)	86.5 (2.33)	86.23 (2.55)	87.73 (3.12)	88.07 (3.18)	89.57 (2.1)
**7 d**	94.3 (3.86)	91.87 (1.65)	92.4 (2.59)	76.77 (2.68)	76.83 (1.23)	73.87 (0.91)	72.67 (2.04)	72.2 (3.16)	73.77 (2.68)	75.1 (2.7)	74.77 (3.15)	71.67 (1.27)
**14 d**	89.63 (2.1)	84 (2.4)	83.83 (2.7)	68.77 (2.59)	68.9 (1.82)	69.5 (2.96)	68.2 (1.85)	66.6 (2.76)	68.4 (2.31)	67.3 (2.69)	64.67 (3.67)	66.83 (5.73)

**Table 4:** Mean (with standard deviation) of expression of integrins and extracellular matrix component (fibronectin, vitronectin, type I collagen) genes of dermal fibroblasts on silver-coated 3D-printed titanium implants S1–S9 following 4, 24, 48, and 72 h of co-culturing. Analysis of gene expression related to fibronectin, vitronectin, and type I collagen was performed at 4 and 72 h after co-culturing cells on the surface of implants.

**α2 Integrin (Collagen-Specific)**									
	**S1**	**S2**	**S3**	**S4**	**S5**	**S6**	**S7**	**S8**	**S9**
**4 h**	1.27 (0.09)	1.15 (0.09)	1.2 (0.08)	1.3 (0.07)	1.22 (0.03)	1.3 (0.1)	1.3 (0.03)	1.23 (0.1)	1.26 (0.13)
**24 h**	1.35 (0.2)	1.36 (0.07)	1.38 (0.06)	2 (0.07)	2.07 (0.08)	2.13 (0.05)	1.77 (0.08)	1.48 (0.06)	1.17 (0.07)
**48 h**	1.65 (0.14)	1.74 (0.06)	1.64 (0.03)	2.37 (0.05)	3.63 (0.3)	4.1 (0.1)	4.14 (0.08)	3.71 (0.3)	3.13 (0.13)
**72 h**	2.21 (0.12)	2.8 (0.1)	2.76 (0.25)	2.96 (0.08)	3.87 (0.22)	5.05 (0.06)	4.82 (0.1)	4.21 (0.16)	3.8 (0.36)
**α5 Integrin (Fibronectin-Specific)**									
	**S1**	**S2**	**S3**	**S4**	**S5**	**S6**	**S7**	**S8**	**S9**
**4 h**	1.08 (0.06)	1.31 (0.08)	1.3 (0.07)	1.33 (0.08)	1.12 (0.11)	1.22 (0.15)	1.46 (0.04)	1.42 (0.02)	1.28 (0.04)
**24 h**	2.12 (0.25)	1.33 (0.06)	1.29 (0.17)	1.99 (0.06)	1.91 (0.02)	2.28 (0.16)	2.22 (0.12)	1.66 (0.12)	1.48 (0.04)
**48 h**	2.28 (0.09)	2.12 (0.08)	1.91 (0.02)	2.25 (0.07)	3.08 (0.06)	3.53 (0.32)	3.65 (0.14)	3.15 (0.05)	2.78 (0.18)
**72 h**	2.99 (0.1)	2.8 (0.27)	2.84 (0.13)	3.27 (0.24)	4 (0.34)	5.34 (0.37)	5.03 (0.06)	4.38 (0.25)	4 (0.11)
**αV Integrin (Vitronectin-Specific)**									
	**S1**	**S2**	**S3**	**S4**	**S5**	**S6**	**S7**	**S8**	**S9**
**4 h**	1.2 (0.18)	1.08 (0.08)	1.22 (0.06)	1.21 (0.12)	1.32 (0.12)	1.4 (0.1)	1.17 (0.19)	1.05 (0.07)	1.22 (0.06)
**24 h**	1.45 (0.15)	1.18 (0.05)	1.3 (0.11)	1.7 (0.13)	2.01 (0.03)	2.36 (0.38)	2.71 (0.08)	1.91 (0.09)	1.69 (0.11)
**48 h**	2.31 (0.17)	1.78 (0.1)	1.69 (0.08)	1.9 (0.1)	3.55 (0.09)	4.03 (0.15)	4.11 (0.1)	3.71 (0.51)	3.27 (0.22)
**72 h**	2.41 (0.17)	2.7 (0.27)	2.86 (0.14)	3.1 (0.54)	4.16 (0.13)	5.2 (0.08)	4.97 (0.06)	4.07 (0.29)	3.42 (0.23)
**β1 Integrin**									
	**S1**	**S2**	**S3**	**S4**	**S5**	**S6**	**S7**	**S8**	**S9**
**4 h**	0.84 (0.13)	0.77 (0.08)	0.9 (0.08)	1.08 (0.1)	1.14 (0.09)	1.08 (0.05)	1.29 (0.02)	1.11 (0.15)	1.05 (0.07)
**24h**	1.04 (0.09)	1.12 (0.11)	1.26 (0.15)	1.14 (0.09)	1.34 (0.05)	1.56 (0.23)	1.59 (0.02)	1.4 (0.02)	1.2 (0.12)
**48 h**	1.22 (0.17)	1.2 (0.01)	1.08 (0.1)	1.11 (0.08)	1.49 (0.11)	2.02 (0.03)	2.16 (0.03)	1.79 (0.06)	1.57 (0.06)
**72 h**	1.41 (0.09)	1.25 (0.15)	1.26 (0.1)	1.3 (0.02)	1.6 (0.26)	2.27 (0.1)	1.91 (0.09)	1.58 (0.19)	1.54 (0.23)
**Vitronectin**									
	**S1**	**S2**	**S3**	**S4**	**S5**	**S6**	**S7**	**S8**	**S9**
**1 d**	1.39 (0.12)	1.9 (0.13)	1.68 (0.05)	1.79 (0.21)	1.77 (0.05)	2.23 (0.05)	2.07 (0.1)	1.85 (0.13)	1.86 (0.17)
**7 d**	3.22 (0.06)	3.24 (0.13)	3.39 (0.03)	3.5 (0.09)	4.09 (0.08)	5 (0.11)	5.05 (0.06)	4.21 (0.17)	4.05 (0.13)
**Fibronectin**									
	**S1**	**S2**	**S3**	**S4**	**S5**	**S6**	**S7**	**S8**	**S9**
**1 d**	1.38 (0.04)	1.54 (0.07)	1.68 (0.05)	1.87 (0.05)	1.53 (0.05)	1.99 (0.06)	1.67 (0.17)	1.64 (0.18)	1.42 (0.04)
**7 d**	2.87 (0.12)	2.81 (0.06)	2.94 (0.07)	3.13 (0.05)	4.06 (0.06)	5.18 (0.21)	5.12 (0.32)	4.55 (0.07)	3.91 (0.12)
**Type I Collagen**									
	**S1**	**S2**	**S3**	**S4**	**S5**	**S6**	**S7**	**S8**	**S9**
**1 d**	1.24 (0.14)	1.39 (0.03)	1.83 (0.07)	2.26 (0.06)	2.5 (0.07)	2.6 (0.19)	2.33 (0.21)	2.08 (0.11)	1.79 (0.21)
**7 d**	3.39 (0.04)	3.51 (0.09)	3.56 (0.22)	3.89 (0.11)	4.44 (0.27)	4.96 (0.03)	5.03 (0.09)	4.33 (0.05)	3.84 (0.51)

**Table 5:** Mean (with standard deviation) of gene (FAK, vinculin, paxillin) expression for MC3T3-E1 cells co-cultured on silver-coated titanium implants with various pore sizes (S1–S9) after 1, 3, 7, and 14 days.

**Paxillin**									
	**S1**	**S2**	**S3**	**S4**	**S5**	**S6**	**S7**	**S8**	**S9**
**1 d**	1.19 (0.02)	1.33 (0.05)	1.11 (0.09)	1.43 (0.17)	1.55 (0.06)	1.4 (0.12)	1.27 (0.15)	1.06 (0.05)	1.11 (0.07)
**3 d**	2.24 (0.12)	2.56 (0.16)	3.11 (0.08)	4.16 (0.1)	4.05 (0.2)	3.3 (0.12)	3.07 (0.1)	2.55 (0.4)	2.68 (0.29)
**7 d**	3.76 (0.1)	3.94 (0.07)	4.76 (0.05)	4.97 (0.11)	5.53 (0.28)	4.33 (0.34)	3.14 (0.14)	2.86 (0.08)	2.59 (0.06)
**14 d**	4.82 (0.13)	5.05 (0.06)	5.88 (0.1)	5.98 (0.08)	6.23 (0.11)	5.45 (0.3)	4.33 (0.34)	4.15 (0.23)	4.25 (0.23)
**FAK**									
	**S1**	**S2**	**S3**	**S4**	**S5**	**S6**	**S7**	**S8**	**S9**
**1 d**	1.07 (0.05)	1.15 (0.05)	0.99 (0.06)	1.08 (0.14)	0.8 (0.13)	0.91 (0.07)	0.82 (0.08)	0.7 (0.23)	0.9 (0.05)
**3 d**	2.12 (0.04)	2.3 (0.07)	2.48 (0.05)	2.34 (0.16)	2.2 (0.1)	1.89 (0.09)	2 (0.24)	1.71 (0.28)	1.79 (0.2)
**7 d**	3.11 (0.09)	3.26 (0.07)	3.54 (0.07)	3.54 (0.04)	3.41 (0.23)	2.43 (0.24)	2.21 (0.64)	2.14 (0.23)	2.04 (0.12)
**14 d**	4.18 (0.1)	4.36 (0.15)	4.65 (0.06)	5 (0.1)	4.78 (0.13)	4.21 (0.16)	3.73 (0.39)	3.15 (0.06)	3.21 (0.2)
**Vinculin**									
	**S1**	**S2**	**S3**	**S4**	**S5**	**S6**	**S7**	**S8**	**S9**
**1 d**	0.43 (0.06)	0.56 (0.14)	0.71 (0.08)	1.07 (0.07)	0.85 (0.11)	1.04 (0.06)	0.65 (0.08)	0.75 (0.08)	0.85 (0.11)
**3 d**	0.89 (0.04)	1.09 (0.08)	1.19 (0.09)	2.17 (0.1)	2.05 (0.06)	1.72 (0.29)	1.17 (0.19)	1.1 (0.03)	1.25 (0.08)
**7 d**	1.5 (0.08)	1.68 (0.06)	1.97 (0.13)	3.05 (0.06)	3.14 (0.14)	2.83 (0.12)	2.07 (0.07)	1.5 (0.15)	1.56 (0.09)
**14 d**	2.62 (0.03)	2.8 (0.03)	3.97 (0.13)	4.15 (0.15)	4.94 (0.13)	4.57 (0.42)	3.2 (0.08)	2.99 (0.04)	2.98 (0.43)

**Table 6:** Mean (with standard deviation) gene expression profiles of osteocalcin, osteopontin, and osteocalcin genes of MC3T3-E1 cells following co-incubation with silver-coated titanium implants (S1–S9) after 1, 3, 7, and 14 days. Analysis for TGF-β1 and SMAD4 gene expression in MC3T3-E1 osteoblast cells was performed on days 1 and 7 after co-incubation.

**Osteocalcin**									
	**S1**	**S2**	**S3**	**S4**	**S5**	**S6**	**S7**	**S8**	**S9**
**1 d**	1.13 (0.22)	0.99 (0.05)	0.85 (0.07)	1.19 (0.13)	1.57 (0.18)	1.33 (0.55)	1.02 (0.11)	0.92 (0.03)	0.89 (0.12)
**3 d**	2.04 (0.08)	2.36 (0.14)	3.23 (0.08)	3.41 (0.2)	4.08 (0.12)	2.76 (0.07)	2.05 (0.11)	1.95 (0.06)	1.79 (0.22)
**7 d**	3.75 (0.06)	4.1 (0.11)	4.42 (0.07)	5.18 (0.1)	5.54 (0.29)	3.31 (0.12)	2.83 (0.1)	2.75 (0.19)	2.76 (0.18)
**14 d**	4.72 (0.1)	5.04 (0.17)	6.2 (0.19)	6.47 (0.26)	7.22 (0.15)	5.17 (0.16)	4.52 (0.3)	4.28 (0.1)	4.32 (0.13)
**Osteopontin**									
	**S1**	**S2**	**S3**	**S4**	**S5**	**S6**	**S7**	**S8**	**S9**
**1 d**	1.03 (0.1)	1.25 (0.1)	1.41 (0.11)	1.99 (0.06)	2.11 (0.1)	1.99 (0.22)	1.11 (0.07)	0.94 (0.01)	0.91 (0.2)
**3 d**	2.63 (0.12)	2.92 (0.12)	4.89 (0.17)	6.16 (0.22)	6.5 (0.38)	5.16 (0.15)	3.59 (0.48)	4.07 (0.05)	3.18 (0.11)
**7 d**	5.81 (0.08)	6.18 (0.13)	8.42 (0.26)	9.14 (0.13)	10.8 (0.31)	8.37 (0.59)	5.9 (0.35)	6.07 (0.12)	5.16 (0.16)
**14 d**	9.05 (0.07)	9.6 (0.17)	11.8 (0.38)	12.75 (0.27)	13.35 (0.59)	12.29 (0.7)	10.33 (0.55)	9.25 (0.28)	7.65 (0.55)
**Osteonectin**									
	**S1**	**S2**	**S3**	**S4**	**S5**	**S6**	**S7**	**S8**	**S9**
**1 d**	1.28 (0.09)	1.28 (0.1)	1.31 (0.11)	1.28 (0.17)	1.66 (0.25)	1.45 (0.07)	1.14 (0.06)	1.08 (0.05)	1.07 (0.09)
**3 d**	2.63 (0.23)	2.9 (0.05)	3.04 (0.08)	4.14 (0.12)	4.12 (0.11)	3.8 (0.62)	2.64 (0.3)	2.6 (0.21)	2.42 (0.06)
**7 d**	3.96 (0.09)	4.2 (0.08)	4.95 (0.08)	5.6 (0.22)	5.5 (0.29)	4.84 (0.17)	3.58 (0.44)	3.44 (0.43)	3.42 (0.22)
**14 d**	5.71 (0.54)	6.21 (0.08)	7.29 (0.1)	7.53 (0.43)	7.7 (0.37)	5.89 (0.44)	4.75 (0.4)	4.8 (0.45)	5.36 (0.1)
**TGF-β1**									
	**S1**	**S2**	**S3**	**S4**	**S5**	**S6**	**S7**	**S8**	**S9**
**1 d**	2.45 (0.08)	2.38 (0.2)	3.22 (0.08)	3.53 (0.32)	3.32 (0.16)	2.79 (0.19)	2.76 (0.08)	2.69 (0.05)	2.56 (0.1)
**7 d**	2.68 (0.1)	2.71 (0.15)	2.84 (0.06)	4.08 (0.08)	4.27 (0.1)	2.95 (0.15)	2.91 (0.03)	2.91 (0.05)	2.85 (0.1)
**SMAD-4**									
	**S1**	**S2**	**S3**	**S4**	**S5**	**S6**	**S7**	**S8**	**S9**
**1 d**	0.8 (0.04)	0.74 (0.13)	0.87 (0.06)	1.01 (0.07)	1.13 (0.08)	1.01 (0.02)	0.92 (0.08)	0.87 (0.07)	0.92 (0.1)
**3 d**	1.6 (0.08)	1.71 (0.05)	2 (0.1)	2.74 (0.23)	3.25 (0.25)	2.75 (0.27)	1.96 (0.1)	1.86 (0.1)	1.83 (0.1)

## Data Availability

The datasets used and/or analyzed during the current study are available from the corresponding authors Maxim Shevtsov and Mark Pitkin on reasonable request.

## Abbreviations

ALP	alkaline phosphatase
ATP	adenosine triphosphate
BMD	bone mineral density
BMP	bone morphogenic protein
CaP	calcium phosphate
CFU	colony-forming units
COL-1	type I collagen
CT	computed tomography
DNA	deoxyribonucleic acid
DSA	direct skeletal attachment
EBM	electron beam melting
ECM	extracellular matrix
ERK	extracellular signal-regulated kinase
FAK	focal adhesion kinase
Fosl1	fos-related antigen 1
GAPDH	glyceraldehyde 3-phosphate dehydrogenase
HA	hydroxyapatite
MAPK	mitogen-activated protein kinase
ML-ALD	molecular layering of atomic layer deposition
MTT assay	3-[4,5-dimethylthiazol]-2, 5-diphenylterazolium bromide assay
OCN	octeocalcin
OPG	osteoprotegerin
PCR	polymerase chain reaction
PVD	physical vapor deposition
RANKL	receptor activator of nuclear factor kappa-Β ligand
RNA	ribonucleic acid
RhoA	ras homolog family member A
ROS	reactive oxygen species
RT-PCR	real-time polymerase chain reaction
Runx2	runt-related transcription factor 2
SBIP	skin and bone integrated pylon
SLM	selective laser melting
SMAD4	SMAD family member 4, Mothers against decapentaplegic homolog 4
TGF-β1	transforming growth factor beta
